# Absence of fluctuation and inverted circadian rhythm of blood pressure increase the risk of cognitive dysfunction in cerebral small vessel disease patients

**DOI:** 10.1186/s12883-023-03107-8

**Published:** 2023-02-15

**Authors:** Yiwen Xu, Chen Gong, Jingxian Liao, Zhonglin Ge, Yu Tan, Yi Jiang, Mengqian Liu, Wen Zhong, Xianxian Zhang, Nan Dong, Xiaozhu Shen

**Affiliations:** 1 Department of Geriatrics, Lianyungang Hospital affiliated to Jiangsu University (Lianyungang Second People’s Hospital), Lianyungang, 222000 China; 2 Department of Neurology, Lianyungang Second People’s Hospital, Lianyungang, China; 3 Department of Geriatrics, Lianyungang Hospital affiliated to Bengbu Medical College (Lianyungang Second People’s Hospital), Lianyungang, China; 4 Department of Neurology, The Sixth Affiliated Hospital of Nantong University, Yancheng Third People’s Hospital, Yancheng, China; 5 Department of Neurology, Suzhou Industrial Park Xinghai Hospital, Suzhou, China

**Keywords:** Blood pressure, Circadian rhythm, Cerebral small vessel disease, Cognitive dysfunction

## Abstract

**Background and purpose:**

Cerebral small vessel disease (CSVD) is a common cause of stroke and senile vascular cognitive impairment, imposing a heavy burden on public health care systems worldwide. Hypertension and 24-hour blood pressure variability (BPV), known to be significant risk factors for cognitive dysfunction, have been found to be associated with cognitive function in CSVD patients in previous studies. However, as a derived part of BPV, there are few studies on the relationship between circadian rhythm of blood pressure and cognitive dysfunction in CSVD patients, and the relationship between them is still unclear. Thus, this study aimed to investigate whether the disturbance of circadian rhythm of blood pressure can affect the cognitive function of patients with CSVD.

**Methods:**

A total of 383 CSVD patients hospitalized in the Geriatrics Department of the Lianyungang Second People’s Hospital between May 2018 and June 2022 were enrolled in this study. The clinical information and parameters of 24-hour ambulatory blood pressure monitoring were compared between the cognitive dysfunction group (*n* = 224) and the normal group (*n* = 159). Finally, a binary logistic regression model was used to assess the relationship between circadian rhythm of blood pressure and cognitive dysfunction in patients with CSVD.

**Results:**

(1) Patients in the cognitive dysfunction group were older, had lower blood pressure on admission, and had a greater number of previous cardiovascular and cerebrovascular diseases (*P* < 0.05). (2) More patients in the cognitive dysfunction group had circadian rhythm abnormalities in blood pressure, especially the non-dipper and reverse-dipper types (*P* < 0.001). (3) In the elderly, there was a statistical difference in the circadian rhythm of blood pressure between the cognitive dysfunction group and the normal group, but this phenomenon did not exist in the middle-aged. (4) Binary logistic regression analysis showed that after adjusting for confounding factors, the risk of cognitive dysfunction in CSVD patients with non-dipper type was 4.052 times higher than that of dipper type (95% CI, 1.782–9.211; *P* = 0.001), and reverse-dipper type was 8.002 times higher than those with dipper type (95% CI, 3.367–19.017; *P*<0.001).

**Conclusions:**

The disturbance of circadian rhythm of blood pressure may affect the cognitive function of patients with CSVD, and the risk of cognitive dysfunction in non-dipper and reverse-dipper types are higher.

## Introduction

Circadian rhythms originated from the evolutionary needs to maximize the fitness of daily organisms on a 24-h timescale by enabling organisms to produce anticipatory and adaptive responses to recurrent light-dark cycles and associated environmental changes [1]. Among them, day-night variations in blood pressure (BP) and heart rate are the most well-known circadian rhythms of physiology [2]. Both systolic blood pressure (SBP) and diastolic blood pressure (DBP) have circadian rhythms that repeat every 24 h in healthy humans. Healthy individuals experience a 10–20% decrease in blood pressure during the night, which is called dipping pattern. Circadian defects in 24-h BP patterns include non-dipper, extreme-dipper and reverse-dipper type [3].

Cerebral small vessel disease (CSVD) is a disorder of the micro-vessels in the brain, which shows various lesions in pathological examination or brain imaging with MRI or CT. Although the underlying pathogenesis remains unclear, CSVD is a common cause of stroke and senile vascular cognitive impairment, which contributes to 45% of dementia cases [4].

Dementia is the leading cause of disability in people over 65 years of age worldwide, including in China, and leads to a heavy burden on public healthcare systems worldwide [5]. Vascular dementia ranks second among all types of dementia, and is very common in elderly patients. Hypertension is known to alter the structure and function of cerebral vessels, and thus hypertension has emerged as a leading cause of age-related cognitive impairment. Previous studies have shown that optimal blood pressure management and control can prevent deterioration of cognitive function, while decreased blood pressure may also affect cognitive function by causing cerebral hypoperfusion [6, 7].

In addition, as a subtype of cerebrovascular disease, the management of cerebrovascular risk factors also plays an important role in the treatment of CSVD, especially in the management of blood pressure. Furthermore, higher 24-hour blood pressure variability (BPV) was found to be associated with CSVD burden [8]. Li XF et al. [9] found that better BP management could reduce the prevalence of cognitive dysfunction in patients with CSVD.

Although many previous studies have examined the effects of hypertension and ambulatory blood pressure changes on cognitive function in CSVD [9–11], there are very few studies on the circadian rhythm of blood pressure and cognitive dysfunction caused by CSVD, and the relationship between them is still unclear. Therefore, this study aimed to analyze the influence of circadian rhythm of blood pressure on cognitive function in patients with CSVD, so as to provide a reference for prevention of cognitive dysfunction in CSVD patients.

## Materials and methods

### Study population

A total of 428 patients with CSVD treated at the Geriatrics Department of the Lianyungang Second People’s Hospital between May 2018 and June 2022 were selected. Eligible participants were recruited based on the following inclusion criteria: age ≥ 35 years old and the diagnosis of CSVD refers to the Chinese consensus on diagnosis and therapy of cerebral small vessel disease 2021 [12], brain MRI showed that the presence of at least one type of white matter hyperintensities (WMH), enlarged perivascular spaces, lacunar infarcts, subcortical infarcts, and cerebral microbleeds. Exclusion criteria were as follows: (1) history of cerebral trauma, surgery, tumor or radiation; (2) combined with severe heart disease such as myocardial infarction, congestive heart failure or severe arrhythmia; (3) history of neuropsychiatric disorders, depression or antidepressants; (4) severe infection or septic shock; (5) liver or renal failure; (6) unable to complete the Mini-Mental State Examination (MMSE) score; (7) missing or incomplete clinical data.

**Fig. 1:**
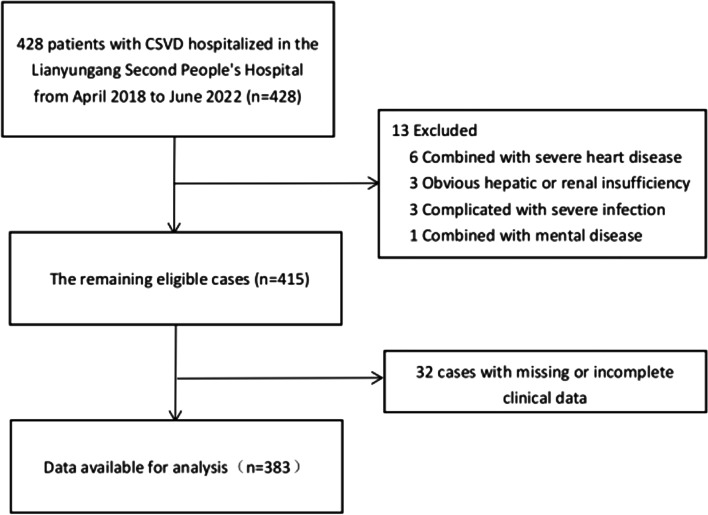
Flow diagram of included and excluded patients. Abbreviations: CSVD, cerebral small vessel disease

Among the 428 patients, 13 met the exclusion criteria, 32 had incomplete or missing data, and 383 eligible patients were finally selected for the study, as shown in Fig. 1. The age range of enrolled patients was 35 to 103 years, with an average age of 73.55 ± 12.44 years, including 171 males (44.6%) and 212 females (55.4%). After obtaining the written informed consent from the enrolled patients, we divided the patients into cognitive dysfunction group (*n* = 224) and the normal group (*n* = 159) according to whether or not the patients had cognitive impairment, for statistical analysis. In addition, if participants in this study were illiterate, written informed consent from the patients’ legal guardians must be obtained.

All supporting data in this study are available from the corresponding author on reasonable request. Ethical approval for this study was obtained from the ethics committee of Lianyungang Second People’s Hospital (No. 2020021).

### Data collection

Baseline clinical information for all enrolled patients was collected from the database, including age, sex, educational level, MMSE score, SBP, DBP, glucose, history of hypertension, diabetes mellitus, prior stroke or TIA, cardiac disease, current smoking (any usage of cigarette per day in the past 30 days) and drinking (drinking more than 100 ml (alcohol content > 50%) per day on average and drinking for more than 1 year; abstaining from drinking for more than 1 year is not). Laboratory findings included alanine aminotransferase, aspartate transaminase, urea nitrogen, creatinine, uric acid, albumin, total cholesterol, triglycerides, low-density lipoprotein cholesterol, high-density lipoprotein cholesterol, Lipoprotein a and C-reactive protein. In addition, we added carotid intima-media thickness based on carotid artery ultrasonography.

The cognitive function of enrolled patients was assessed by MMSE within 48 h after the diagnosis of CSVD, and each patient was evaluated face-to-face by an experienced neurologist. A MMSE score of < 27 was used as the cut-off value of cognitive dysfunction.

All patients underwent 24-h ambulatory blood pressure monitoring (ABPM) (MedLifeKC-2820, China) within 48 h after diagnosis of CSVD, which were performed by trained nurses in accordance with the requirements of the 2020 Chinese Hypertension League Guidelines on Ambulatory Blood Pressure Monitoring. In the devices used, 7:00–21:00 was set as day-time and 21:00–7:00 was set as night-time. BP was measured every half hour during day-time and every hour during night-time. Patients were asked to get adequate rest or sleep during the night-time and maintain normal activities during the day-time. Daily activities and sleep and wake times were needed to be recorded in a diary. Patients were allowed to take part in daily activities during blood pressure monitoring. The criteria of effective blood pressure were: 40–255 mmHg for SBP, 10–195 mmHg for DBP, 40–240 bpm for heart rate. After wearing the device for at least 24 h, the patients removed it and the data from it was downloaded for analysis. Then relevant blood pressure parameters were collected, such as 24-h mean systolic blood pressure, 24-h mean diastolic blood pressure, 24-h mean standard deviation of systolic blood pressure, 24-h mean standard deviation of diastolic blood pressure, daytime mean systolic blood pressure (dmSBP), daytime mean diastolic blood pressure (dmDBP), nighttime mean systolic blood pressure (nmSBP), nighttime mean diastolic blood pressure (nmDBP). And then we calculated the ratios of night systolic and diastolic blood pressure reduction separately: the ratios of night systolic blood pressure reduction = (dmSBP-nmSBP)÷dmSBP× 100%, the ratios of night diastolic blood pressure reduction = (dmDBP-nmDBP)÷dmDBP× 100%. We defined the ratios of night systolic blood pressure reduction as the ratios of night blood pressure reduction (ΔMBP) when the blood pressure circadian rhythm shown by the ratios of night systolic and diastolic blood pressure reduction was inconsistent. Next, the circadian rhythms of blood pressure were classified according to ΔMBP: 0–10% was non-dipper type, 10–20% was dipper type, more than 20% was extreme-dipper type and less than 0% was reverse-dipper type.

### Statistical analysis

Data were analyzed using the SPSS software (IBM SPSS Statistics for Windows, version 26.0; IBM Corp., Armonk, NY, USA) and graphics were drawn using GraphPad Software (GraphPad Prism for Windows, version 9.0.0; San Diego, CA, USA). Baseline information included clinical parameters, medical history, biochemical variable and ambulatory blood pressure parameters. Kolmogorov-Smirnov test were used to assess the normality of numerical variables. Mann-Whitney U test were used for analysis in the case of non-normal distribution, described by median and interquartile range (IQR). The continuous variables of normal distribution were analyzed by independent sample T-test and expressed as mean ± standard deviation (SD), and chi-squared test or Fisher exact test for categorical variables. In addition, we used binary logistic regression models to assess the association between circadian blood pressure and cognitive dysfunction in patients with cerebral small vessel disease. A 2-tailed *P* value< 0.05 was considered significant.

## Results

### Baseline characteristics

From May 2018 to June 2022, 383 eligible CSVD patients (44.6% male, mean age 74 years) were finally selected for this study. All patients were divided into the cognitive dysfunction group (*n* = 224) and the normal group (*n* = 159) by MMSE score. Next, Table 1 summarizes the baseline clinical and biochemical characteristics of the two groups.

**Table 1:** Baseline clinical characteristics and biochemical indicators between the cognitive dysfunction group and the normal group

	Overall (*n* = 383)	Cognitive dysfunction group (*n* = 224)	Normal group (*n* = 159)	*P* value
**Clinical parameters**				
Age, Mean (SD)—year	73.55 ± 12.44	78.77 ± 10.82	66.19 ± 10.76	< 0.001^a^
Sex—no. (%)				0.144^b^
Male	171(44.6%)	93(41.5%)	78(49.1%)	
Female	212(55.4%)	131(58.5%)	81(50.9%)	
Educational level—no. (%)				0.338^b^
illiteracy	88(23.0%)	52(23.2%)	36(22.6%)	
Primary school education	72(18.8%)	41(18.3%)	31(19.5%)	
Junior high school education	79(20.6%)	49(21.9%)	30(18.9%)	
High School education	92(24.0%)	58(25.9%)	34(21.4%)	
undergraduate college	52(13.6%)	24(10.7%)	28(17.6%)	
MMSE score, Median (IQR)	25.00(21.00, 28.00)	21.50(15.00, 24.00)	28.00(28.00, 29.00)	< 0.001^c^
SBP, Mean (SD)—mmHg	148.26 ± 24.90	146.10 ± 23.74	151.30 ± 26.22	0.044^a^
DBP, Mean (SD)—mmHg	83.31 ± 15.55	81.37 ± 16.67	86.05 ± 13.40	0.003^a^
Glucose, Median (IQR)—mmol/L	6.62(5.65, 8.05)	6.70(5.73, 8.25)	6.60(5.63, 7.83)	0.449^c^
**Medical history**				
Hypertension—no. (%)	306(79.9%)	174(77.7%)	132(83.0%)	0.199^b^
Diabetes mellitus—no. (%)	92(24.0%)	50(22.3%)	42(26.4%)	0.355^b^
TIA or stroke—no. (%)	137(35.8%)	97(43.3%)	40(25.2%)	< 0.001^b^
cardiac disease—no. (%)	131(34.2%)	86(38.4%)	45(28.3%)	0.040^b^
smoking—no. (%)	68(17.8%)	34(15.2%)	34(21.4%)	0.117^b^
drinking—no. (%)	51(13.3%)	20(8.9%)	31(19.5%)	0.003^b^
antiplatelet drugs—no. (%)	228(59.5%)	136(60.7%)	92(57.9%)	0.575^b^
antihypertensive drugs—no. (%)	298(77.8%)	171(76.3%)	127(79.9%)	0.412^b^
hypoglycemic drugs—no. (%)	88(23.0%)	46(20.5%)	42(26.4%)	0.178^b^
antihyperlipidemics—no. (%)	273(71.3%)	162(72.3%)	111(69.8%)	0.593^b^
**Laboratory indicators**				
ALT, Median (IQR)—U/L	25.00(19.00,32.00)	24.00(18.00,30.00)	26.00(22.00,36.00)	< 0.001^c^
AST, Median (IQR)—U/L	24.00(21.00,28.00)	23.00(20.00,28.00)	25.00(21.00,29.00)	0.178^c^
BUN, Median (IQR)—mmol/L	6.60(5.30,8.30)	6.90(5.40,8.90)	6.30(5.22,7.27)	0.004^c^
Cr, Median (IQR)—umol/L	70.00(58.00,89.00)	74.85(61.00,94.00)	65.00(55.00,80.00)	0.001^c^
UA, Median (IQR)—umol/L	324.00(272.00,397.50)	336.33(273.00,410.60)	316.00(266.00,374.00)	0.092^c^
ALB, Median (IQR)—g/L	41.40(37.90,44.60)	40.20(36.70,43.08)	43.20(40.60,45.30)	< 0.001^c^
TC, Median (IQR)—mmol/L	4.67(3.95,5.27)	4.65(3.86,5.19)	4.67(4.17,5.38)	0.064^c^
LDL-C, Median (IQR)—mmol/L	2.89(2.30,3.38)	2.85(2.20,3.33)	2.89(2.41,3.50)	0.035^c^
HDL-C, Median (IQR)—mmol/L	1.19(1.01,1.35)	1.20(1.01,1.36)	1.19(1.03,1.34)	0.763^c^
TG, Median (IQR)—mmol/L	1.63(1.15,2.21)	1.58(1.12,1.99)	1.77(1.22,2.46)	0.050^c^
Lp(a), Median (IQR)—mg/L	171.00(73.00,288.00)	190.00(80.00,266.75)	157.00(68.00,314.00)	0.597^c^
CRP, Median (IQR)—mg/L	2.07(0.61,6.28)	2.49(0.65,6.28)	1.71(0.54,6.28)	0.094^c^
**Carotid artery ultrasonography**				
CIMT, Mean (SD)—mm	0.79 ± 0.14	0.80 ± 0.14	0.77 ± 0.13	0.054^a^

*Abbreviations*: *SD* Standard deviation, *IQR* Interquartile range, *MMSE* The Mini-Mental State Examination, *SBP* Systolic blood pressure, *DBP* Diastolic blood pressure, *ALT* Alanine aminotransferase, *AST* Aspartate transaminase, *BUN* Urea nitrogen, *Cr* Creatinine, *UA* Uric acid, *ALB* Albumin, *TC* Total cholesterol, *LDL-C* Low-density lipoprotein cholesterol, *HDL-C* High-density lipoprotein cholesterol, *TG* Total glyceride, *Lp(a)* Lipoprotein a, *CRP C*-reactive protein, *cIMT* Carotid intima-media thickness

^a^Analyzed by independent sample T-test

^b^Analyzed by chi-squared test

^c^Analyzed by Mann-Whitney U test

Compared to the normal group, patients in the cognitive dysfunction group were older, had lower MMSE score, baseline SBP and DBP. In terms of past medical history, not surprisingly, patients with cognitive impairment were more likely to have had cardiovascular or cerebrovascular diseases than those with normal cognitive function. As for biochemical variables, patients with cognitive dysfunction had higher urea nitrogen, creatinine, and lower alanine aminotransferase and albumin. There was no difference in gender, education level, glucose, other medical history and Laboratory indicators between the two groups (all *P* > 0.05).

### Ambulatory blood pressure parameters

**Fig. 2:**
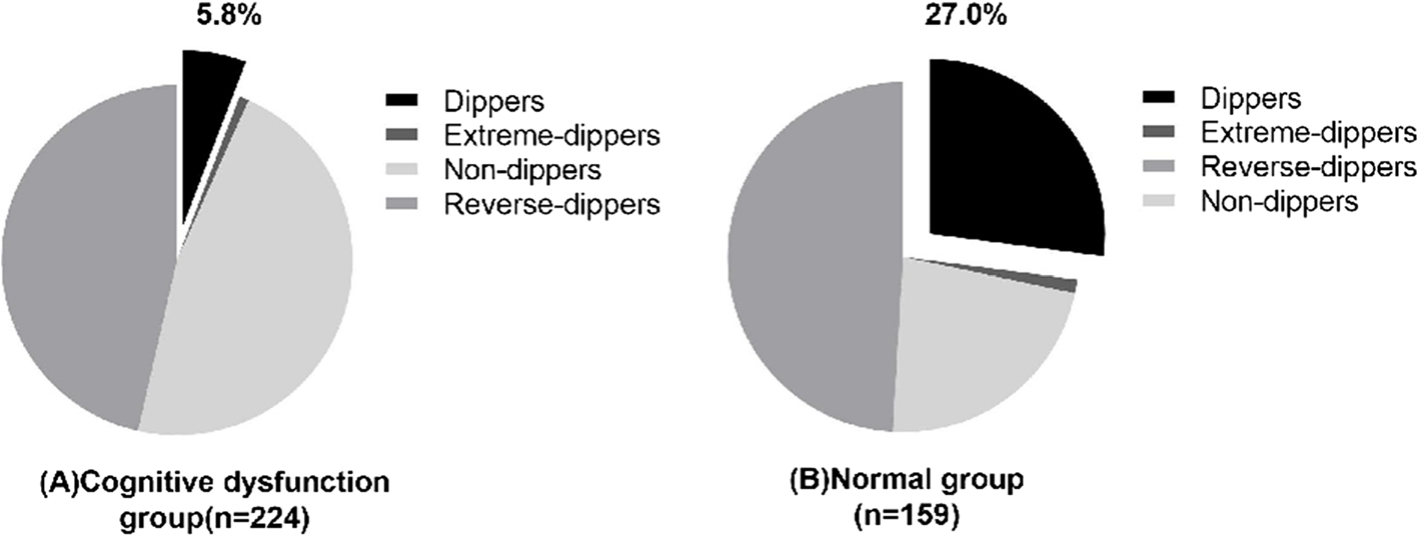
Pie charts of circadian rhythm of blood pressure in two groups

As expected, more patients in the cognitive dysfunction group had circadian abnormalities in blood pressure, especially the non-dipper and reverse-dipper types. In contrast, compared with the cognitive dysfunction group, more patients in the normal group had dipper type (27.0% vs. 5.8%), as shown in Fig. 2. Furthermore, patients with cognitive impairment had lower 24-h mean diastolic blood pressure and dmDBP, but higher nmSBP, as shown in Table 2.

**Table 2:** Ambulatory blood pressure parameters between the cognitive dysfunction group and the normal group

Characteristics of blood pressure	Overall (*n* = 383)	Cognitive dysfunction group (*n* = 224)	Normal group (*n* = 159)	*P* value
Circadian rhythm of blood pressure—no. (%)				
Abnormal*	337(85.8%)	211(94.2%)	116(73.0%)	< 0.001^a^
Dippers	56(14.6%)	13(5.8%)	43(27.0%)	< 0.001^a^
Extreme-dippers	4(1.0%)	2(0.9%)	2(1.3%)	
Non-dippers	183(47.8%)	105(46.9%)	78(49.1%)	
Reverse-dippers	140(36.6%)	104(46.4%)	36(22.6%)	
24hmSBP, Mean (SD)—mmHg	136.97 ± 19.77	137.17 ± 19.30	136.69 ± 20.47	0.818^b^
24hmDBP, Mean (SD)—mmHg	73.91 ± 12.23	72.04 ± 11.64	76.53 ± 12.60	< 0.001^b^
24hSBP-SD, Mean (SD)—mmHg	16.72 ± 4.52	16.71 ± 4.74	16.72 ± 4.21	0.979^b^
24hDBP-SD, Mean (SD)—mmHg	11.36 ± 3.75	10.95 ± 3.92	11.93 ± 3.44	0.011^b^
dmSBP, Mean (SD)—mmHg	137.89 ± 20.08	137.18 ± 19.84	138.89 ± 20.45	0.413^b^
dmDBP, Mean (SD)—mmHg	74.78 ± 12.81	72.44 ± 11.81	78.09 ± 13.45	< 0.001^b^
nmSBP, Mean (SD)—mmHg	135.12 ± 21.35	137.72 ± 20.33	131.47 ± 22.27	0.005^b^
nmDBP, Mean (SD)—mmHg	71.70 ± 12.48	70.87 ± 12.07	72.87 ± 12.98	0.122^b^

*Abbreviations*: *SD* Standard deviation, *24hmSBP* 24-hour mean systolic blood pressure, *24hmDBP* 24-hour mean diastolic blood pressure, *24hSBP-SD* 24-hour mean standard deviation of systolic blood pressure, *24hDBP-SD* 24-hour mean standard deviation of diastolic blood pressure, *dmSBP* Daytime mean systolic blood pressure, *dmDBP* Daytime mean diastolic blood pressure, *nmSBP* Nighttime mean systolic blood pressure, *nmDBP* Nighttime mean diastolic blood pressure

^a^Analyzed by chi-squared test

^b^Analyzed by independent sample T-test

*Defined as the sum of extreme-dippers, non-dippers and reverse-dippers

Next, we also stratified the two groups according to whether or not they were older than 60 years old. Table 3 presents that in the elderly patients, the circadian rhythm of blood pressure was statistically different between the cognitive dysfunction group and the normal group, but this phenomenon was not present in the middle-aged patients.

**Table 3:** Ambulatory blood pressure parameters of the two groups, stratified by age

Circadian rhythm of blood pressure	Age ≤ 60			*P* value	Age>60			*P* value
	Overall (*n* = 58)	Cognitive dysfunction group (*n* = 11)	Normal group (*n* = 47)		Overall (*n* = 325)	Cognitive dysfunction group (*n* = 213)	Normal group (*n* = 112)	
Abnormal^a^	40 (69.0%)	7 (63.6%)	33 (70.2%)	0.950	287 (88.3%)	204 (95.8%)	83 (74.1%)	<0.001
Dippers	18 (31.0%)	4 (36.4%)	14 (29.8%)	0.412	38 (11.7%)	9 (4.2%)	29 (25.9%)	<0.001
Extreme-dippers	1 (1.7%)	0 (0.0%)	1 (2.1%)		3 (0.9%)	2 (0.9%)	1 (0.9%)	
Non-dippers	27 (46.6%)	3 (27.3%)	24 (51.1%)		156 (48.0%)	102 (47.9%)	54 (48.2%)	
Reverse-dippers	12 (20.7%)	4 (36.4%)	8 (17.0%)		128 (39.4%)	100 (46.9%)	28 (25.0%)	

^a^Defined as the sum of extreme-dippers, non-dippers and reverse-dippers

### Circadian rhythm of blood pressure and cognitive dysfunction

We then used circadian rhythm of blood pressure as a categorical variable to investigate the relationship between circadian rhythm of blood pressure and cognitive dysfunction in patients.

In general, as shown in Table 4, abnormal circadian rhythm of blood pressure is associated with cognitive dysfunction in patients with CSVD. Further to say, taking dipper type as a reference, non-dipper type and reverse-dipper types were closely related to cognitive dysfunction.

**Table 4:** Association between circadian rhythm of blood pressure (as a categorical variable) and cognitive dysfunction

		B	SE	WaldX^2^	*P* value	OR	95% CI
Model 1	Dippers (reference)			37.618	<0.001	1.000	
	Extreme-dippers	1.196	1.049	1.301	0.254	3.308	0.423 ~ 25.843
	Non-dippers	1.494	0.350	18.205	<0.001	4.453	2.242 ~ 8.842
	Reverse-dippers	2.257	0.371	37.032	<0.001	9.556	4.619 ~ 19.768
Model 2	Dippers (reference)			23.524	<0.001	1.000	
	Extreme-dippers	2.162	1.089	3.942	0.047	8.687	1.028 ~ 73.395
	Non-dippers	1.270	0.397	10.220	0.001	3.562	1.635 ~ 7.759
	Reverse-dippers	1.985	0.418	22.537	<0.001	7.281	3.208 ~ 16.526
Model 3	Dippers (reference)			22.299	<0.001	1.000	
	Extreme-dippers	1.485	1.340	1.227	0.268	4.414	0.319 ~ 61.063
	Non-dippers	1.399	0.419	11.149	0.001	4.052	1.782 ~ 9.211
	Reverse-dippers	2.080	0.442	22.172	<0.001	8.002	3.367 ~ 19.017

*Abbreviation*s: *B* Regression coefficient, *SE* Standard error,*WaldX*^*2*^ Chi-square value, *OR* Odds ratio, *CI* Confidence interval

Model 1: unadjusted

Model 2: adjusted for age, drinking, history of cardiac disease and TIA or stroke

Model 3: additionally adjusted for alanine aminotransferase, urea nitrogen, creatinine, albumin and low-density lipoprotein cholesterol

Univariate analysis showed that the influence of circadian rhythm classification of blood pressure on cognitive dysfunction was statistically significant in Model 1 (*P*<0.001), which still existed after we adjusted for confounding factors in Model 2 and Model 3. We can see from Model 3 that the risk of cognitive dysfunction in CSVD patients with non-dipper type was 4.052 times higher than that of dipper type (95% CI, 1.782–9.211; *P* = 0.001), and reverse-dipper type was 8.002 times higher than those with dipper type (95% CI, 3.367–19.017; *P*<0.001).

## Discussion

According to the statement by the Lancet Commission on dementia prevention, intervention and care, approximately 50 million people were living with dementia in 2020, and this number is expected to rise to 152 million by 2050 [13]. Adjusted for age and gender, the overall prevalence of dementia in China is estimated to be 6.0%, with 15.07 million dementia patients aged 60 years or older [14].

CSVD represents a group of diseases with heterogeneous etiology and pathological mechanisms that affect the small arteries, arterioles, venules, and capillaries of the brain [15]. The effects of small vessel disease on brain parenchyma are mainly lesions located in subcortical structures, such as white matter hyperintensities, enlarged perivascular spaces, lacunar infarcts, subcortical infarcts, cerebral microbleeds and brain atrophy, which can often be detected by brain imaging techniques [15, 16]. Small vessel disease has an important role in cerebrovascular disease, and is a leading cause of cognitive decline and functional loss in the elderly.

Once cognitive dysfunction is allowed to progress to the dementia stage, much of the damage is irreversible. Therefore, early detection and intervention of cognitive dysfunction is particularly important. It is well known that dementia and cognitive impairment share similar risk factors, among which vascular factors are easily detected by screening. Therefore, effective control of vascular factors such as hypertension and dyslipidemia can delay the decline of cognitive function and reduce the occurrence of dementia [13, 14]. In addition to average blood pressure, fluctuations in blood pressure are known to affect brain health to some extent. Recent studies have found that BPV is a vascular factor associated with the risk of CSVD progression and dementia, and also affects cognitive function in the elderly [17–19]. Li C et al. [20] found that higher long-term BPV in middle-aged and elderly is associated with accelerated cognitive decline in a non-linear dose-response relationship. A systematic review and meta-analysis by Rianne et al. concluded that BPV was associated with dementia and cognitive impairment, and the relative contribution of BPV exceeded that of mean blood pressure [21]. Circadian rhythm of blood pressure is one of the main evaluation indicators of BPV, which can be obtained by 24-h ABPM in major medical institutions. 24-h circadian rhythm disorders are known to be common in older adults. Research evidence suggest that disruption of circadian function may be an early manifestation of neurodegeneration and a risk factor for neurodegenerative diseases in older healthy adults, including Alzheimer’s disease and related dementia [22, 23].

Although abnormal circadian rhythms of blood pressure have been recommended as an important cardiovascular risk factor by some national guidelines for hypertension management [24], few studies have examined the relationship between circadian rhythms of blood pressure and cognitive function. The role of abnormal circadian rhythm of blood pressure in CSVD patients with cognitive dysfunction remains unclear. Previous studies have found that the effect of elevated blood pressure on cognitive decline is associated with vascular pathology, nerve plaques and neurofibrillary tangles, which suggests that controlling blood pressure can alleviate vascular and neurodegenerative pathways [25, 26]. Hence, it is necessary to comprehensively monitor the circadian rhythm of blood pressure in CSVD patients in order to prevent and delay the occurrence of cognitive impairment. Therefore, we designed this study to analyze the relationship between circadian rhythm of blood pressure and cognitive function in CSVD patients by grouping them according to their cognitive status and recording the instantaneous blood pressure at admission and 24-h ABPM data of each patient.

A retrospective cohort study found that nocturnal blood pressure rise was associated with reduced cognitive performance and could reduce MMSE by 2.9%, which can be used as a predictor of cognitive impairment among the elderly [27]. Takahiro et al. [28] further found a strong correlation between the reverse-dipper circadian rhythm of blood pressure and cognitive dysfunction in the elderly. Komori et al. [29] quantitatively measured WMH volumes and found that the WMH volume increased 2.4 times in patients with non-dipper type of nocturnal BP compared to those with normal dipper type, which is similar to the findings of Hua Zhang et al. [24]. As a distinctive feature of human CSVD, WMH can be observed around the ventricles and subcortical areas. Researchers have proposed several hypotheses for the pathogenesis of white matter lesions (WML). Among them [30, 31], demyelinating injury secondary to hypoperfusion and blood-brain barrier leakage due to pathological vasodilation mediated by endothelial cells dysfunction are considered to be the most important causes of WML. The major risk factors for WML progression are age and hypertension [32, 33]. Hypertension leads to atherosclerotic plaque formation, thickening of the tunica media and narrowing of the lumen, and these vessels are usually derived from cortical and leptomeningial arteries that nourish the deep white matter. Besides, it also increases vascular fibrosis and changes the distribution of extracellular matrix, leading to the hardening of vessel walls and the reduction of CBF [34, 35]. Furthermore, subcortical arteriolar occlusion can affect multiple cognitive domains, thereby increasing the risk of vascular cognitive impairment [35]. In early-stage hypertension, non-dipping type is often associated with microvascular damage rather than macrovascular damage [36]. Thus, we speculate that the reason might be that the high level of nocturnal blood pressure will lead to long-term state of high load in the cerebrovascular vessels, resulting in cerebral microvascular endothelial damage, and accelerating the progress of atherosclerosis, leading to cerebral hypoperfusion, further damaging the white matter of the brain, and eventually leading to cognitive dysfunction.

In this study, we found that the number of patients with abnormal circadian rhythm of blood pressure in the cognitive dysfunction group was higher than that in the normal group, especially among the elderly patients. Next, we included all enrolled patients in bivariate logistic regression models and found that the non-dipper and reverse-dipper types were at greater risk of cognitive dysfunction, which was consistent with their findings. Furthermore, we also found in Table 4 that even when we adjusted the factor of age in model 2 and model 3, it had no effect on the subsequent results. Thus, we believe that the circadian rhythm of blood pressure plays a major role in this study.

In summary, abnormal circadian rhythm of blood pressure is a predictor of cardiovascular and cerebrovascular diseases independent of blood pressure level and has important clinical significance. However, different types of abnormal circadian rhythm of blood pressure may lead to significant differences in the risk of cognitive dysfunction in CSVD patients. This reminds us that medical professionals should actively restore and maintain the normal circadian rhythm of blood pressure in patients with CSVD while reducing blood pressure steadily in the early stage, which is critical for preventing target organ damage and improving the prognosis of patients. In addition, since each patient has a different type of blood pressure circadian rhythm, we suggest that anti-hypertensive regimens should be individually selected, and attention should be paid to the influence of the time of taking anti-hypertensive drugs on the different types of circadian rhythms of blood pressure.

Nevertheless, this study had some limitations. First, as a single-center retrospective study, some selection bias might exist in the process of data collection and analysis. In addition, since this was a cross-sectional study, the causal relationship could not be determined, so further large-scale studies are needed. Second, the current study population was Asian Chinese, but CSVD may be influenced by genetic factors, thus the results of this study may not be applicable to other ethnic groups. Third, about 80% of the patients in this study had a history of hypertension, so the influence of different types of antihypertensive drugs on the circadian rhythm of blood pressure cannot be ruled out. Finally, due to database limitations, we were unable to collect the Montreal Assessment of Cognitive Function for each patient and were unable to classify patients with mild cognitive impairment.

## Conclusion

The disturbance of circadian rhythm of blood pressure may affect the cognitive function of patients with CSVD, and the risk of cognitive dysfunction in non-dipper and reverse-dipper types are higher.

## Data Availability

The datasets used and/or analyzed during the current study are available from the corresponding author on reasonable request.
